# “Nil per oral after midnight”: Is it necessary for clear fluids?

**DOI:** 10.4103/0019-5049.71044

**Published:** 2010

**Authors:** Kajal S Dalal, Dhanwanti Rajwade, Ragini Suchak

**Affiliations:** BARC Hospital, Anushakti Nagar, Mumbai - 400 094, India

**Keywords:** Clear fluids, preoperative fasting, pulmonary aspiration, stomach contents - pH, volume

## Abstract

Fasting before general anaesthesia aims to reduce the volume and acidity of stomach contents, thus reducing the risk of regurgitation and aspiration. Recent guidelines have recommended a shift in fasting policies from the standard ‘nil per oral from midnight’ to a more relaxed policy of clear fluid intake a few hours before surgery. The effect of preoperative oral administration of 150 ml of water 2 h prior to surgery was studied prospectively in 100 ASA I and II patients, for elective surgery. Patients were randomly assigned to two groups. Group I (n = 50) was fasting overnight while Group II (n = 50) was given 150 ml of water 2 h prior to surgery. A nasogastric tube was inserted after intubation and gastric aspirate was collected for volume and pH. The gastric fluid volume was found to be lesser in Group II (5.5 ± 3.70 ml) than Group I (17.1 ± 8.2 ml) which was statistically significant. The mean pH values for both groups were similar. Hence, we conclude that patients not at risk for aspiration can be allowed to ingest 150 ml water 2 h prior to surgery.

## INTRODUCTION

Long fasting hours prior to surgery is a great discomfort to the patient. Despite recent guidelines stating that it is appropriate to reduce the interval of clear fluid ingestion to 2 h prior to surgery,[1] it is common practice to follow “nothing by mouth” or Nulla per os (NPO) after midnight for both solids as well as clear fluids. Decreasing the fasting period enhances the quality and efficiency of anaesthesia care by decreasing the cost, increasing the patient satisfaction and avoiding delays and cancellations. Also there is a decrease in the risk of dehydration and hypoglycaemia and thereby decrease in the perioperative morbidity.

Previous studies have shown that pH< 2 and volume of gastric aspirate > 25 ml (0.4 ml/kg) predispose a patient to pulmonary aspiration,[2] hence a strict overnight fasting regimen was instituted. However, the cochrane database has reviewed several studies showing that prolonged withholding of oral fluids does *not* improve gastric pH or volume, and permitting a patient to drink fluids preoperatively may even result in significantly lower gastric fluid volumes.[3] In an attempt to reduce the fasting hours of a patient preoperatively without increasing the risk of pulmonary aspiration, we decided to assess the safety of ingestion of 150 ml of water 2 h prior to surgery in patients undergoing general anaesthesia with endotracheal intubation.

## METHODS

After Ethics Committee approval with written informed consent, 100 ASA I and II patients between 12 and 60 years of age, posted for elective orthopaedic, gynaecological, otolaryngological and general surgery were divided into two groups. Emergency surgeries, patients with history of acid peptic disease, anticipated difficult intubation, diabetes mellitus, obesity, pregnancy, hiatus hernia[4] as well as those routinely taking any medications that affected gastric motility or secretion were excluded from the study.

Group I was kept fasting overnight whereas Group II was given 150 ml water 2 h prior to surgery. Patients were premedicated with midazolam and pentazocine, and general anaesthesia was induced using intravenous thiopentone sodium followed by vecuronium. An 18 G and 16 G Ryle’s tube was inserted in male and female patients, respectively after intubation and its position was confirmed by auscultation over the epigastrium for insufflated air. Gastric aspirate was obtained through a 20 ml syringe with the patient supine with an assistant massaging the upper abdomen, as well as with various other positions like Trendelenburg, left lateral and right lateral positions to facilitate maximal aspiration.

Volume of aspirate was noted and pH measured using a standardized pH strip. Sex, age, weight, type of surgery, duration of fasting and interval between ingestion of water and surgery was documented. Results were given as mean ± SD. Data collected were analysed using Student’s t-test. Differences were considered statistically significant if *P* values were <0.05.

## RESULTS

There was no significant difference between the groups with regard to weight, age and sex. Patients who were kept fasting overnight (Group I) had an average fasting time of 12 h. The ingestion - surgery interval for Group II was on an average 2 h [Table 1].

**Table 1:** Patient demographics

	Group I	Group II
Age (years)	42 ± 12.96	44 ± 16.42
Males	18	32
Females	32	27
Weight (kg)	51 ± 9.21	53 ± 7.84
Ingestion-surgery interval (min)	742 ± 70.08	130 ± 6.64

Except for sex, values are expressed as Mean ± SD

Patients who had 150 ml of water (Group II) had lesser volume of gastric aspirate (5.5 ± 3.70 ml) than that of Group I (17.1 ± 8.21 ml) which was statistically significant [Table 2]. The pH was found to be in the same range for both the groups (Group I: 1.7 ± 0.28, Group II: 1.6 ± 0.26) [Table 2]. Patients at high risk i.e. gastric fluid volume > 25 ml and pH <2.5 are shown in Table 3. Group I had four patients with a combination of both risk factors, while none were present in Group II.

**Table 2:** Comparison of volume and pH of gastric fluid in both groups

	Group I	Group II
pH	1.7 ± 0.28	1.6 ± 0.26
Extremes	1.5, 2.5	1.5, 2.5
Gastric fluid volume (ml)	17.1 ± 8.21	5.5 ± 3.70+
Extremes (ml)	5, 42	2, 18

+ *P* value <0.05

**Table 3:** Incidence of risk factors

	Group I	Group II
Volume > 25 ml	4	0
pH < 2.5	29	29
Volume > 25 ml and pH < 2.5	4	0

## DISCUSSION

Pulmonary aspiration of gastric contents during anaesthesia though a *rare* event,[5] with an incidence of 1 in 7,000 to 8,000 in ASA I and II patients, and 1 in 400 ASA III to V patients,[6] is still considered a significant cause of anaesthesia-related deaths. The severity of pulmonary damage is related to both the volume and pH of the gastric fluid. A combination of volume > 25 ml and pH < 2.5 is considered lethal.[2] Hence any safety measure that *reduces* this hazard is preferred, so the routine preoperative practice of “nothing by mouth after midnight” is followed. But unfortunately, the ‘nil per oral’ order is blindly applied to both liquids and solids and has become engrained in our anaesthetic practice.[7]

The time required for solid food to liquefy and enter the small intestine depends on the type of food ingested (being shorter for carbohydrates and proteins than for fats and cellulose) and the food particle size.[8] Complete emptying of solids from the stomach takes 3 to 6 h, but may be prolonged by fear, pain or opioids.[9] So it is appropriate that no solid food be eaten on the day of surgery. However, the gastro-oesophageal emptying of liquids is rapid wherein studies have shown that gastric emptying after intake of a carbohydrate drink is complete within 2 h of ingestion.[10]

At the time of induction of anaesthesia, gastric fluid volume is quite variable in normal people. Even if the patient is fasting, the stomach is not totally empty. On an average, 25 ml to 35 ml of gastric fluid remains in the stomach.[6] Comparing this to the traditional cut-off of gastric fluid volume >25 ml and pH < 2.5, 30-60% patients would be at a risk of pulmonary aspiration, but on an average, the incidence is as low as 1 in 3000.[11] Passive *regurgitation* of gastric contents can occur only if intragastric pressure exceeds the protective tone of the lower oesophageal sphincter, and for pulmonary *aspiration* to occur, the protective airway reflexes must also be abolished.[6]

Our study was undertaken to determine whether a 2 h fast with clear fluids was safe for patients. Clear fluids would include black tea, coffee, water, carbonated drinks and fruit juices without any particulate matter.[12] We chose 150 ml of water to be given 2 h prior to surgery. We used a Ryle’s tube for aspiration of gastric contents which is a well accepted method for assessment.[5,6,13,14] Our study confirmed the results of previous studies[3,5,6] that even after 11-13 h of fasting, a large number of patients had gastric pH < 2.5 and gastric fluid volume >25 ml.

Patients who received 150 ml water actually had decreased gastric fluid volume which was statistically significant as seen in another study.[3] The pH remained unaffected, thereby not increasing the risk of pulmonary complications due to aspiration. Studies have also shown that giving clear fluids increased patient comfort, decreased anxiety and thirst.[10,15]

We conclude that it is safe to conduct general anaesthesia in patients who have ingested 150 ml of water 2 h prior to surgery. Prolonged withholding of oral fluid does not decrease gastric fluid volume and pH. Clinicians should appraise this evidence and adopt the recent ASA guidelines which recommend an evolution from the indiscriminate ‘NPO after midnight’ blanket fasting policy. However, the customary 8 h fasting should be followed for patients at a higher risk of aspiration like in diabetes mellitus, pregnancy, obesity, etc. as more research is necessary to determine the safety in these patients. The risk of unexpected regurgitation cannot be avoided even by overnight fasting, and anaesthesiologists must always be prepared to deal with these complications.
